# The Association of Combined *GSTM1* and *CYP2C9* Genotype Status with the Occurrence of Hemorrhagic Cystitis in Pediatric Patients Receiving Myeloablative Conditioning Regimen Prior to Allogeneic Hematopoietic Stem Cell Transplantation

**DOI:** 10.3389/fphar.2017.00451

**Published:** 2017-07-11

**Authors:** Chakradhara Rao S. Uppugunduri, Flavia Storelli, Vid Mlakar, Patricia Huezo-Diaz Curtis, Aziz Rezgui, Yves Théorêt, Denis Marino, Fabienne Doffey-Lazeyras, Yves Chalandon, Peter Bader, Youssef Daali, Henrique Bittencourt, Maja Krajinovic, Marc Ansari

**Affiliations:** ^1^Onco-Hematology Unit, Geneva University Hospital, Department of Pediatrics Geneva, Switzerland; ^2^CANSEARCH Research Laboratory, Department of Pediatrics, Faculty of Medicine, University of Geneva Geneva, Switzerland; ^3^Clinical Pharmacology and Toxicology Service, Geneva University Hospital Geneva, Switzerland; ^4^CHU Sainte-Justine Research Center, Charles-Bruneau Cancer Center, Montreal QC, Canada; ^5^Clinical Pharmacology Unit, CHU Sainte-Justine, Montreal QC, Canada; ^6^Division of Hematology, Department of Medical Specialties, Geneva University Hospital Geneva, Switzerland; ^7^Division for Stem Cell Transplantation and Immunology, University Hospital Frankfurt Frankfurt, Germany; ^8^Department of Pediatrics, Charles-Bruneau Cancer Center, CHU Sainte-Justine Research Center, Montreal QC, Canada

**Keywords:** busulfan, cyclophosphamide, acrolein, HepaRG, urothelial cells, induction, CYPs, conjugation

## Abstract

Hemorrhagic cystitis (HC) is one of the complications of busulfan-cyclophosphamide (BU-CY) conditioning regimen during allogeneic hematopoietic stem cell transplantation (HSCT) in children. Identifying children at high risk of developing HC in a HSCT setting could facilitate the evaluation and implementation of effective prophylactic measures. In this retrospective analysis genotyping of selected candidate gene variants was performed in 72 children and plasma Sulfolane (Su, water soluble metabolite of BU) levels were measured in 39 children following treatment with BU-CY regimen. The cytotoxic effects of Su and acrolein (Ac, water soluble metabolite of CY) were tested on human urothelial cells (HUCs). The effect of Su was also tested on cytochrome P 450 (CYP) function in HepaRG hepatic cells. Cumulative incidences of HC before day 30 post HSCT were estimated using Kaplan–Meier curves and log-rank test was used to compare the difference between groups in a univariate analysis. Multivariate Cox regression was used to estimate hazard ratios with 95% confidence intervals (CIs). Multivariate analysis included co-variables that were significantly associated with HC in a univariate analysis. Cumulative incidence of HC was 15.3%. In the univariate analysis, HC incidence was significantly (*p* < 0.05) higher in children older than 10 years (28.6 vs. 6.8%) or in children with higher Su levels (>40 vs. <11%) or in carriers of both functional *GSTM1* and *CYP2C9* (33.3 vs. 6.3%) compared to the other group. In a multivariate analysis, combined *GSTM1* and *CYP2C9* genotype status was associated with HC occurrence with a hazards ratio of 4.8 (95% CI: 1.3–18.4; *p* = 0.02). Ac was found to be toxic to HUC cells at lower concentrations (33 μM), Su was not toxic to HUC cells at concentrations below 1 mM and did not affect CYP function in HepaRG cells. Our observations suggest that pre-emptive genotyping of *CYP2C9* and *GSTM1* may aid in selection of more effective prophylaxis to reduce HC development in pediatric patients undergoing allogeneic HSCT.

**Article summary**:

(1) Children carrying functional alleles in *GSTM1* and *CYP2C9* are at high risk for developing hemorrhagic cystitis following treatment with busulfan and cyclophosphamide based conditioning regimen.

(2) Identification of children at high risk for developing hemorrhagic cystitis in an allogeneic HSCT setting will enable us to evaluate and implement optimal strategies for its prevention.

**Trial registration**: This study is a part of the trail “clinicaltrials.gov identifier: NCT01257854.”

## Introduction

One of the common complications of allogeneic hematopoietic stem cell transplantation (HSCT) in children is the occurrence of hemorrhagic cystitis (HC). HC occurring within 48 h after infusion of cyclophosphamide (CY) as a part of conditioning regimen is referred to as early onset and after 48 h as late onset HC (Russell et al., 1994). HC incidence in an allogeneic HSCT was reported to be as high as 20% in pediatric patients (Gargiulo et al., 2014; Hayden et al., 2015; Lunde et al., 2015). A myeloablative conditioning regimen with busulfan (BU) and CY in children undergoing HSCT is often associated with higher incidences of HC compared to the reduced intensity regimen excluding CY (Seber et al., 1999; Silva Lde et al., 2010). Besides the presence of CY as a component of conditioning, several other risk factors were defined for HC incidence such as viral infections (human BK/JC polyoma virus, Adenovirus, and Cytomegalovirus), unrelated or haploidentical related donor, cord blood transplantation and high doses of BU (Tsuboi et al., 2003). Indeed HC is one of the causes of morbidity especially during the pre-engraftment period usually before day 30 post HSCT impacting the overall survival (OS) in pediatric HSCT with 40% OS observed in patients with HC compared to 65% in those without HC (Cesaro et al., 2008). The incidence of HC can be very high (up to 60%) when CY is administered post HSCT as a prophylaxis for GvHD in haploidentical transplant recipients (Ruggeri et al., 2015), pinpointing the need for identification of high risk patients prior to CY therapy.

**FIGURE 1:**
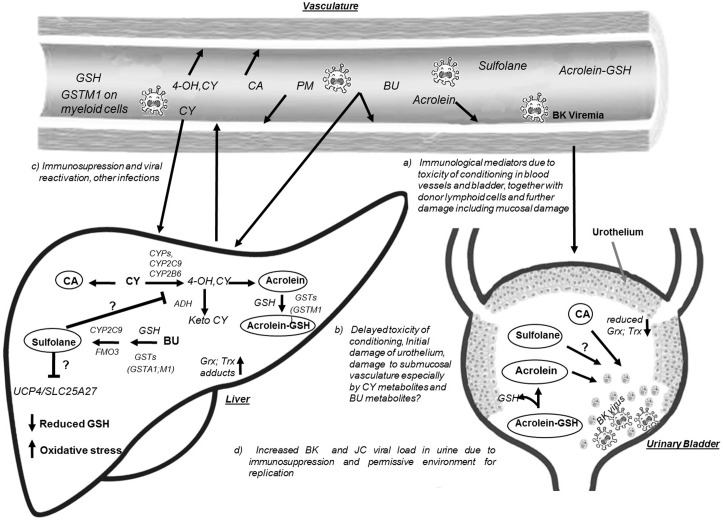
Hypothetical model for Hemorrhagic Cystitis occurrence in children receiving BU-CY regimen prior to allogeneic HSCT. Busulfan (BU) and cyclophosphamide (CY) metabolism predominantly occurs in the liver generating the water soluble metabolites sulfolane (Su) and acrolein (Ac), respectively. During the metabolic process BU utilizes glutathione (GSH) and activity of GSTs such as GST alpha 1 (GSTA1), GST mu1 (GSTM1) plays an important role in conjugation of BU with GSH. GSH is also essential for scavenging the CY active metabolites including Ac also catalyzed by GSTs. When CY is administered after BU, the water soluble metabolite of BU, i.e., Su could influence the activity of cytochrome P 450 enzymes (CYPs) and thus might affect the activation of CY, or on the other hand higher Su levels also represents the utilization of GSH and formation of γ-glutamyl-dehydroalanyl-glycine (EdAG) affecting the availability of reduced form of GSH. Su might also affect the function of GSTs and other enzymes in BU, CY metabolism and proteins such as Mitochondrial uncoupling proteins (UCP) encoded by SLC25A27 gene affecting cell survival. Su possibly affect the function of ADH (alcohol dehydrogenase) enzyme resulting in increased levels of hydroxy CY metabolite, resulting increased Ac formation. BU could irreversibly alkylate the glutaredoxins (Grx) and Thioredoxins (Trx) increasing the oxidative stress, thus increasing toxicity of incoming CY metabolites such as 4 hydroxy cyclophosphamide (4OH-CY); Ac, chloro acetaldehyde (CA), and Phosphoramide mustard (PM). Altered activity of CYPs due to less functional alleles in the encoding genes might have a predictable function of Su formation and CY activation. Circulating CY metabolites (4OH CY, PM) including Ac inhibit cytokine function by alkylation and also cause immunosuppression, further increasing the chances of viremia and viruria (BK and/or JC virus). Ac conjugated with GSH in the liver gets delivered to the bladder and releases free Ac which then could alkylates cysteine residues and causing toxicity. Altogether increased rate of metabolite delivery to the urinary bladder together with viral load and lack of protection due to increased oxidative stress, endothelial damage to the blood vessels, urothelial damage and enhanced viral replication results in bladder inflammation and hematuria and the condition hemorrhagic cystitis.

The pathophysiology of HC occurring 24–72 h after HSCT is multifactorial (Leung et al., 2005), involving initial chemical injury (damage to the bladder transitional epithelium and the endothelium of blood vessels by BU, CY and their metabolites), viral infections with viruria, immunosuppression, poor immune reconstitution, engraftment process, and HC pathogenesis (**Figure 1**). It is well-established that damage to the urothelium is the initial step during pathogenesis of HC. Though it is known that acrolein (Ac, metabolite of CY) contributes to HC development (Conklin et al., 2009), the role of Sulfolane (Su, water soluble metabolite of BU) is not yet known. It remains unclear whether Su affects the activity of enzymes implicated in CY metabolism (CYP2C9, CYP2C19, and CYP2B6) or if it is directly involved in HC pathogenesis. It was hypothesized that BU metabolites may affect the activity of CYPs, thus influencing the outcomes such as increased occurrence of treatment related toxicity with BU-CY compared to CY-BU administration *via* increased exposure to CY metabolites (Cantoni et al., 2011; Rezvani et al., 2013). However, experimental data on the effect of BU metabolites on CYP function is limited. Moreover, the concentration and time dependent effects of Ac on the urothelium are unknown. With the availability of an analytical method for quantifying Su levels in plasma (Versace et al., 2012), it is now possible to investigate its relation to HC incidence in a clinical setting. Several enzymes contribute to the metabolism of Su and CY, for example, CYP2C9 is involved in both the processes of activation of CY and formation of Su from BU (Uppugunduri et al., 2014). Flavin monooxygenase (FMO3) is involved in Su formation, while CYP2B6, CYP2C19, alcohol dehydrogenase (ADH) and aldehyde dehydrogenase (ALDH3A1) are involved in either activation or inactivation of CY (Timm et al., 2005; Xie et al., 2006). The metabolic pathway of CY is outlined in Supplementary Figure S1.

The participation of several glutathione *S* transferase (GST) isoforms in eliminating BU and CY intermediate metabolites also indicates their importance in the etiology of HC, especially isoforms that are predominantly expressed in all tissues including urothelium, such as *GSTM1*. Moreover, variations in these genes may affect enzyme activity that in turn affect CY and BU metabolism contributing to the development of HC. In this retrospective study, the role of Su levels, selected genetic variants in CY metabolism genes, and Su formation were investigated with relation to the incidences of HC. Functional studies with human urothelial cells (HUCs), and human hepatic/hepatoma cells (HepaRG/HepG2) as well as with alcohol dehydrogenase enzyme (ADH) were also conducted to explore the cytotoxic potential of Su, Ac and ADH enzyme modulating effects of Su.

## Materials and Methods

### Study Subjects

This retrospective analysis was conducted in 72 children recruited from St. Justine Hospital (CHUSJ), Montreal, Canada and University Hospital of Geneva (HUG), Geneva, Switzerland between 2001 and 2013. Su levels were measured in 39 patients due to non-availability of plasma from the rest. Local Institutional Ethics Committee at each center approved the study and all patients or their parents signed the informed consent. Details of inclusion criteria are available at Clinicaltrials.gov site (NCT01257854).

### Conditioning Regimen, Measurement of Plasma Levels of Sulfolane, and Hemorrhagic Cystitis Diagnosis Criteria

All patients received i.v. BU (Days -9 to -6 of HSCT) followed by i.v. CY (days -5 to -2 of HSCT). BU was administered by i.v. route in 16 doses as a 2 h infusion every 6 h. BU first dose pharmacokinetic parameters were estimated and adjusted from the 3rd to 6th dose, aiming to achieve a steady state concentration between 600 and 900 ng/mL (Ansari et al., 2014). I.V. CY was administered as a 2 h infusion at 50 mg/kg/day and each patient received a cumulative dose of 200 mg/kg. 2-mercaptoethane sulfonate (MESNA; UROMITEXAN^®^) was administered as a 15 min i.v. infusion at 25 mg/kg/dose, one dose 15 min prior to CY and three doses at 3, 7, and 11 h from the beginning of CY therapy. Patients were hyper hydrated with 3000 mL/m^2^/day during and until 24 h after the end of CY administration. Plasma from patients collected before dose 7 and after dose 9 BU infusions were used for measuring Su levels using gas-chromatography and mass spectrometry (Versace et al., 2012). Plasma levels of Su were normalized with cumulative BU dose in mg/kg (up to dose 6 for before dose 7 Su levels, up to dose 9 for after dose 9 Su levels).

Hemorrhagic cystitis was defined as the presence of hematuria (both microscopic and macroscopic) from the initiation of the conditioning regimen up to 30 days post-transplant, which is usually considered as engraftment period (0–30 days). HC was graded according to the criteria described previously (Hingorani, 2009): grade I = microscopic hematuria, grade II = macroscopic hematuria, grade III = macroscopic hematuria with clots, and grade IV = macroscopic hematuria with clot needing instrumentation for clot evacuation or leading to urinary retention or requiring surgical intervention.

### Genotyping and Gene Expression Analysis

Genomic DNA extracted from peripheral mononuclear cells of patients before they received the conditioning regimen was used for genotyping relevant genetic variants. The list of chosen genetic variants, functionality and the genotyping method used are enlisted in **Table 1**. RNA was extracted from HepaRG cells using PureLink^®^ RNA mini kit (Thermo Fischer Scientific), was converted to cDNA using SuperScript^®^ VILO^TM^ cDNA Synthesis Kit (Thermo Fischer Scientific) and was used for gene expression experiments with gene specific primers (Supplementary Table S1) using SYBR^®^ Green Real-Time PCR Master Mix (Thermo Fischer Scientific). The primers were tested for efficiency at five different levels, and *GAPDH* was used as the internal control gene. TaqMan genotyping and gene expression assays (in triplicates on three different occasions) were performed on StepOnePlus real time PCR system (Thermo Fischer Scientific).

**Table 1:** The list of genetic variants genotyped in the selected genes and the minor allele frequencies observed in the study population.

SNP (rsID)	Nucleotide variation (amino acid change) functional impact in homozygous carriers	Method	Real time PCR assay ID	Minor allele frequency (*n* = 144)	Heterozygosity (*n* = 72)
GSTM1^∗^0	Deletion No enzyme activity	PCR/melt curve SYBR green chemistry	See main text for reference	48.6	____
CYP2C9^∗^2 (rs1799853)	c.430C > T (p.Arg144Cys) Decreased activity	Real time DME Taqman assay	C_25625805_10	12.5	16.7
CYP2C9^∗^3 (rs1057910)	c.1075A > C (p. Ile359Leu) Decreased activity	DME Taqman assay	C_27104892_10	8.3	16.7
CYP2C19^∗^2 (rs4244285)	c.681G > A in exon 5 Splicing defect and no enzyme activity	DME Taqman assay	C_25986767_70	12.5	22.2
CYP2C19^∗^17 (rs12248560)	c.-806C > T and c.-3402C > T (promoter region polymorphism) Increased expression of CYP2C19 and its activity	DME Taqman assay	C_469857_10	19.4	27.8
FMO3^∗^ (rs2266780)	Glu308Gly; missense variation	DME Taqman assay	C__2220257_30	20.8	27.8
FMO3^∗^ (rs2266782)	Glu158Lys; missense variation	DME Taqman assay	C__2461179_30	43.1	38.9
FMO3^∗^ (rs1736557)	Val257Met; missense variation	DME Taqman assay	C__8698544_30	7.6	15.3
CYP2B6^∗^5 (rs3211371)	Arg487Cys; decreased enzyme activity	DME Taqman assay	C_30634242_40	13.9	22.2
CYP2B6^∗^9 (rs3745274)	Gln172His; decreased enzyme activity	DME Taqman assay	C_7817765_60	28.5	29.2
ALDH3A1^∗^2 (rs2228100)	Pro329Ala; decreased activity	Taqman assay	C___7443700_60	34.0	34.7
SLC25A27 (rs9381468)	c.299-346A > G; influence on function or expression not known	Taqman assay	C__29893451_10	45.1	51.4
SLC25A27 (rs953062)	c.299-346A > G; influence on function or expression not known	Taqman assay	C__11541519_20	37.5	45.8

*^∗^Single-nucleotide polymorphisms (SNPs) in FMO3 gene were predicted to be tolerant using prediction tools such as sorting intolerant from tolerant (SIFT) and protein analysis through evolutionary relationships (PANTHER), except rs2266780 that was predicted to be damaging to gene function by SIFT*.

### Human Urothelial (HUC), Hepatoma (HepG2), and Hepatic (HepaRG) Cell Culture

Human urothelial cells (ScienCell Research laboratories) were obtained from Chemie Brunschwig, Basel and were cultured in urothelial cell medium (ScienCell Research laboratories) supplemented with growth factors as per the instructions by the supplier (ScienCell) on poly-L-lysine-coated (0.01% from Sigma–Aldrich) culture vessels (BD Biosciences) at 37°C and 5% CO_2_. HUC Cells were seeded at a density of 5000 cells/cm^2^ and were passaged when reached 80–90% confluence using trypsin EDTA (Lifescience technologies), and all experiments were performed in between passages 2 and 6.

Undifferentiated hepatic cells (HepaRG) were procured from Biopredic^®^ (Saint-Gregoire, France) and were cultured based on the protocol by Antherieu et al. (2010) for stable expression, activity, and inducibility of cytochromes P450 in differentiated HepaRG cells. Undifferentiated HepaRG cells (passage 18) were seeded on day 1 in 75 cm^2^ flasks at a density of 2.6 × 10^4^ cells/cm^2^ in a culture medium composed of Williams medium E, supplemented with 10% fetal bovine serum (FBS), 2 mM Glutamax^TM^, 50 μM hydrocortisone hemisuccinate, 5 μg/mL insulin, 100 IU/mL penicillin, and 100 μg/mL streptomycin at 37°C and 5% CO_2_. Culture medium was changed every 2–3 days. On day 15, cells were confluent and differentiation was induced by adding 1.5% DMSO in the culture medium (differentiation medium). On day 29, differentiated HepaRG cells were detached by gentle trypsinization and plated in 24-wells at a density of 2 × 10^5^ cells/cm^2^ in the same differentiation medium.

HepG2 cell lines (ATCC) were cultured using DMEM medium (Life technologies) supplemented with 10% FBS (AMMED), at 37°C and 5% CO_2._ Experiments were performed at passage numbers 80–95.

### Evaluation of CYP Function and Expression in HepaRG Cells

HepaRG cells preincubated with Su were used for CYP phenotyping and expression analysis. On day 36, differentiation medium was replaced by the induction medium (culture medium deprived of DMSO and FBS). On days 37, 38, and 39 Su was added to the induction medium at concentrations ranging from 0.5 to 5 μM. Rifampicin (10 and 50 μM) and phenobarbital (500 and 1000 μM) were used as positive controls. Final concentration of DMSO during the time of induction was 0.1%. On day 40 after 72 h of induction, cells were washed three times with phenol-red free Williams medium E and a probe cocktail composed of midazolam 5 μM (CYP3A4), *S*-mephenytoin 50 μM (CYP2C19), bupropion 50 μM (CYP2B6), flurbiprofen 10 μM (CYP2C9), and phenacetin 10 μM (CYP1A2) in phenol-red free Williams medium E was incubated for 3 h. The incubation medium was removed and stored at -80°C for further quantification of the metabolites released in the medium. All the cells from triplicate wells were detached by gentle trypsinization (100 μL of trypsin-EDTA 0.05% per well), homogenized by pipetting cell suspension up and down and samples were split into two parts. Cells were then washed twice with phosphate buffer saline. The first half of the cells was lysed with RIPA buffer (Thermo Scientific) for further protein quantification using bicinchoninic acid assay. The other half of cells was used for RNA extraction and cDNA synthesis followed by gene expression analysis. The experiment was repeated twice on different occasions with each exposure in triplicate wells. Though CYP1A2 probe was included in the cocktail, only the results of CYP2C9, CYP2C19, CYP2B6, and CYP3A4 probes are presented in this report.

For phenotyping, incubation medium samples were thawed at room temperature and extracted with ethyl acetate 2:1 (v:v). Hydroxymidazolam-d4, paracetamol-d3, hydroxymephenytoin-d3 and hydroxybupropion-d6 were added as internal standards (33 ng/ml). The organic phase was transferred to a clean tube, evaporated with a vacuum concentrator Mivac^®^ (Genevac, Ipswich, United Kingdom) and then reconstituted with water/acetonitrile (80:20). Metabolites were quantified by high-performance liquid chromatography (HPLC) tandem mass spectrometry (MS). Chromatography was performed in a gradient mode with an Agilent series 1100 (Waldbronn, Germany) LC system composed of a Discovery C18 column (5 μM particle size, 15 cm × 2.1 mm) preceded by a Discovery pre-column (5 μM; 5 cm × 2.1 mm; Supelco^®^, Bellefonte, PA, United States) at 20°C. The HPLC eluents were water + 0.1% formic acid (mobile phase A) and acetonitrile + 0.1% formic acid (mobile phase B).

The tandem mass spectrometry experiments were performed with an API 4000 triple quadrupole mass spectrometer (AB Sciex, Concord, ON, Canada) controlled by Analyst 1.5.1 Software. The mass spectrometer was operated in the multiple reaction monitoring (MRM) mode with both positive and negative electrospray ionizations. Chromatographic conditions following sample injection (10 μl) were as follows: initial conditions were 2% mobile phase B, and then mobile phase B was increased by gradient up to 90% from 0.1 to 3 min. This condition was kept during 1 min and then initial conditions were applied until the end of the run. Mobile phase flow rate was maintained at 0.35 mL/min and chromatography performed at 20°C with the total run time per injection of 10 min for positive mode and 9 min for negative mode. Transitions of the probe drug metabolites quantified by mass spectrometry are described in Supplementary Table S2.

### Evaluation of Alcohol Dehydrogenase (ADH) Inhibition by Sulfolane

Alcohol dehydrogenase inhibition assays were performed as previously described (Jin et al., 2004) with few modifications. In brief, ADH inhibition was assessed using ethanol at 10 mM concentration as a substrate for ADH (Sigma–Aldrich). The enzyme activity catalyzed by ADH was measured by monitoring NADH. The enzyme reaction was started by mixing ethanol, NAD+ and ADH in a buffer. Tecan sunrise spectrophotometer (Tecan) was used to measure the accumulation of NAD+ by measuring absorbance of light at 340 nm. The rate of the reaction was reported as change in the absorbance of light (340 nm) per minute (ΔA340/min). Initially with NAD+ at 1.5 mM and ADH concentration of 0.005 unit/0.2 mL, we observed 8–13 mM as a Michaelis–Menten constant (Km) of ADH with ethanol as substrate. This reaction was linear within a 30-min time range. Further inhibition experiments were conducted using same conditions to measure ADH activity with pre-incubations of pyrazole (known inhibitor of ADH) at 1, 5, 10, 50, 100, 500, and 1000 μM and Su at 0.1, 0.5, 1, 5, 10, 50, and 100 μM concentrations.

### IC50 Screening, Cell Viability, and AnnexinV Assays Following Incubation with Ac or Su

IC50 screening and apoptotic behavior of cells by annexin assay was performed with a series of Ac and Su concentrations. For IC50 screening in HUC cells, 4000 cells were seeded in a poly L- lysine (0.01%) coated 96 well plate followed by incubation with a series of concentrations of either Ac (0.084, 0.84, 4.2, 8.4, 84, 168, 336, and 672 μM) or Su (0.01, 0.1, 1, 10, 100 mM) for about 48 h followed by WST-1 assay (Roche). IC50 values were determined by non-linear curve fitting of absorbance readings at 450 nm against the concentrations. Since both Ac, and Su are water soluble agents, they were easily dissolved in the culture medium to obtain appropriate concentrations. Further, these observations were confirmed by trypan blue exclusion method and microscopical examination of cells.

For AnnexinV binding assays, 125,000 cells were seeded in a 25 cm^2^ flask coated with 0.01% poly-L-lysine. Cells were incubated for 24 h, followed by exposure to either Ac (33, 50, 84, 250, 500, and 830 μM) or Su (0.1 and 1 mM) for 30 and 60 min. After treatment, cells were incubated in fresh medium for 48 h and were harvested by trypsinization (0.05%). Fractions of live, early apoptotic, late apoptotic, and dead cells were measured using Fluorescein isothiocyanate (FITC) Annexin V Apoptosis Detection Kit I (BD pharmingen, BD biosciences) according to manufacturer’s instructions. Flow cytometric analysis was carried out using CyAn ADP (Beckman Coulter) flow cytometer. FITC Annexin V was measured at FL1 (excitation laser: 488 nm; emission filter: 530/40), PI was measured at FL6 (excitation laser: 561 nm, emission filter: 586/15), no compensation was necessary. A minimum of 4000 cells per sample were analyzed. Gates were adjusted based on the same batch of HUC cells which were unlabeled and labeled either by FITC Annexin V or propidium iodide only. Results are given as fraction of live, early apoptotic, late apoptotic and dead cells in the whole cell population.

### Statistical Analysis

Statistical analyses were performed using IBM^®^ SPSS^®^ statistics (version 22, SPSS, Inc., Armonk, NY, United States). Two sided *p*-values were represented and a probability of less than 0.05 was considered as statistically significant. Mann–Whitney *U*-test was used to analyze the differences in demographics between groups of with and without HC. A receiver–operator characteristic curve (ROC) for Su levels, age and weight was plotted to show the trade-off in sensitivity vs. 1- specificity rates for HC, as the cut-off of the test was shifted from low to high. Mann–Whitney *U*-test was used to analyze the relation of Su levels and HC. Cumulative incidences of HC were estimated using Kaplan–Meier curves and log-rank test was used to compare the difference between groups divided based on cutoff defined in ROC curves, in univariate analysis. In univariate analysis the variables included were: age (age above and below 10 years), weight (above and below 30 kg), *CYP2C9* and *GSTM1* combined genotypes (normal vs. variant), *FMO3* (normal vs. variant), other genotypes (Normal vs. variant), source (cord blood vs. bone marrow), aGvHD prophylaxis (Cyclosporine vs. Cyclosporine + steroid vs. cyclosporine+ methotrexate) diagnosis (malignant vs. non-malignant), donor type (matched related, matched unrelated, mismatched related, and mismatched unrelated) and serotherapy (received vs. not received). Multivariate analysis was performed including significant co-variates in univariate analysis using Cox (Proportional Hazards) regression to estimate hazard ratios (HR) with 95% confidence intervals (CIs). As there is a correlation between age and weight, in the multivariate analysis, we used only age as a co-variate. Multivariate analysis with Su levels was performed including either dose 7 or dose 9 levels together with age. Competing risk-analysis was not performed as the competing events such as non-relapse mortality and relapse occurred only after HC occurrence (only one patient) or not occurred before day 30 post-transplant. IC50 values for Ac and Su were calculated using non-linear regression using GraphPad Prism version 6.0 for Windows (GraphPad Software, La Jolla, CA, United States^1^) with a correlation coefficient set to a minimum of 0.85 and including at least 20 points for curve fitting. The CYP activity phenotyping results are presented as a fold change compared to that of control group.

## Results

The demographic and clinical characteristics of patients with and without HC are outlined in **Table 2**. The overall demographic and transplant characteristics are summarized in Supplementary Table S3 and clinical outcomes other than HC are summarized in **Table 3**. Cumulative incidence of HC was observed to be 15.3%, median time to occurrence of HC was 23 days and to resolve HC was 32 days post HSCT. BK or JC viruria was detected in 9 patients tested out of 11 with HC and thus the cause could be attributed mainly to their presence. As for the remaining, etiology of HC was unknown. Seven out of eleven patients with HC (63.6%) engrafted before the occurrence of HC. Please refer to **Table 4** for clinical characteristics of patients with HC.

**Table 2:** Demographic and transplant characteristics of the patients included in the study.

		39 Children with Su levels		Total 72 Children	
Characteristic *N* (%)		With HC^∗^	Without HC	With HC^¥^	Without HC
Gender	Male	4 (21.1)	15 (78.9)	6 (16.7)	30 (83.3)
	Female	4 (20.0)	16 (80.0)	5 (13.8)	31 (86.2)
Diagnosis	Malignant disease	6 (26.1)	17 (73.9)	9 (20.5)	35 (79.5)
	Non-malignancy	2 (12.5)	14 (87.5)	2 (7.1)	26 (92.9)
Source	Bone marrow	2 (11.1)	16 (88.9)	3 (9.7)	28 (90.3)
	Cord blood	6 (28.5)	15 (71.5)	8 (19.5)	33 (80.5)
HLA match	MRD	1 (7.1)	13 (92.9)	2 (7.4)	25 (92.6)
	MMRD	0	3 (100)	0	3 (100)
	MUD	1 (16.6)	5 (83.4)	1 (7.7)	12 (92.3)
	MMUD	6 (37.5)	10 (62.5)	8 (27.6)	21 (72.4)
Serotherapy	ATG (yes/no)	6/2 (75/25)	24/7 (77.4/22.6)	8/3 (72.7/27.3)	47/14 (77.1/22.9)
GvHD prophylaxis	Cyclosporine	0	1 (100)	0	2 (100)
	Cyclosporine+steroids	5 (25.0)	15 (75.0)	7 (18.4)	31 (81.6)
	Cyclosporine+ methotrexate	3 (16.7)	15 (83.3)	4 (12.5)	28 (87.5)

**Characteristic**		**Median (mean)**			

Age (years)		14.5 (12.6)	5.6 (6.2)	12.7 (15.0)	5.6 (6.8)
Weight (kg)		45.4 (44.9)	21.5 (23.8)	49.2 (45.3)	21.5 (25.8)
BU cumulative dose in mg/kg		14.5 (14.7)	16.1 (16.2)	13.9 (14.8)	15.9 (16.0)
CY cumulative dose in mg/kg		200	200	200	200
Su levels before dose 7 (ng/mL//mg/kg)^1^		50.7 (48.3)	34.4 (35.1)	NA	NA
Su levels after dose 9 (ng/mL//mg/kg)^2^		40.7 (40.5)	29.9 (30.9)	NA	NA

^∗^*Six of them were positive for BK virus and one patient for JC virus in urine. Age, weight, Su (sulfolane) levels differed significantly between the two groups (*p* < 0.05). All of them received MESNA as prophylaxis. ^¥^Eight of them were positive for BK virus and one for JC virus in urine. ^1,2^Sulfolane levels were normalized with the BU dose received till that level in mg/kg. HC, hemorrhagic cystitis; MRD, matched related donor; MMRD, mismatched related donor; MUD, matched unrelated donor; MMUD, mismatched unrelated donor*.

**Table 3:** Cumulative incidences of clinical outcomes other than hemorrhagic cystitis.

Clinical outcomes	Cumulative incidence		Day of onset	

	***N***	**Percentage**	**Median**	**Range**
Neutrophil recovery(Day 100)	64	88.8	18	10–48
Platelet recovery (Day 180)	59	81.9	42	16–173
Sinusoidal obstruction syndrome (SOS)	7	9.7	11	4–26
Acute graft vs. host disease (grades 2–4)	9	12.5	95	30–162
Death	14	19.4	183.5	28–1259
Relapse^∗^	11	25.0	163	44–384
Rejection	7	9.7	45	33–246

^∗^*Frequencies are calculated for malignancies alone*.

**Table 4:** Clinical characteristics of patients with hemorrhagic cystitis.

Patient no	Age (Y)	Weight (Kg)	Sex	Diagnosis	Donor source	Etiology of HC	*CYP2C9* diplotype	*GSTM1* genotype	HC grade	Day of onset of HC	Day when HC was resolved^∗^	Day of neutrophil engraftment^∗^
HC1	6.5	25.0	M	MDS	CB	BK	^∗^1^∗^1	NonNull	3	25	39	15
HC2	16.8	52.8	M	MPD	BM	BK	^∗^1^∗^1	NonNull	1	28	40	31
HC3	3.1	14.3	F	AML	CB	BK	^∗^1^∗^2	NonNull	2	23	32	18
HC4	16.8	65.3	M	AML	CB	BK	^∗^1^∗^1	NonNull	2	24	129	19
HC5	5.2	24.1	M	AML	CB	BK	^∗^1^∗^1	NonNull	3	23	38	14
HC6	17.1	49.2	F	AML	BM	unknown	^∗^1^∗^1	NonNull	2	3	7	18
HC7	13.9	56.1	M	MDS	CB	JC	^∗^1^∗^1	Null	2	3	9	No engraftment
HC8	19.9	77.5	F	Immunodefficiency	CB	BK	^∗^1^∗^1	NonNull	3	27	47	34
HC9	15.0	64.2	F	AML	CB	unknown	^∗^1^∗^1	NonNull	1	18	22	43
HC10	15.1	38.0	M	Immunodefficiency	BM	BK	^∗^1^∗^1	NonNull	3	20	30	16
HC11	10.2	31.3	F	ALL	CB	BK	^∗^1^∗^1	Null	2	11	13	14

^∗^*Days post-hematopoietic stem cell transplantation. ALL, acute lymphoblastic leukemia; AML, acute myeloid leukemia; F, female; BK virus, human polyoma virus named after the patient initials in which it was isolated first. CB, cord blood; BM, bone marrow; HC, hemorrhagic cystitis; JC virus, John Cunningham human polyoma virus. M, male; MDS, myelodysplastic syndrome; MPD, myelo proliferative disorder; Virus were detected in urine (viruria); grade I = microscopic hematuria; grade II = macroscopic hematuria; grade III = macroscopic hematuria with clots, and grade IV = macroscopic hematuria with clot needing instrumentation for clot evacuation or leading to urinary retention or requiring surgical intervention*.

### Sulfolane Levels, Age, and HC

**FIGURE 2:**
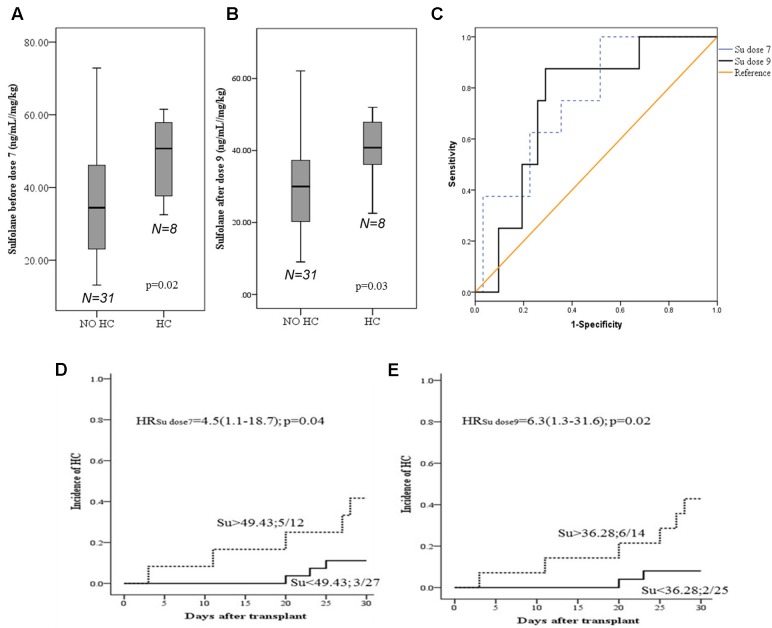
Sufolane levels and occurrence of Hemorrhagic cystitis. **(A,B)** Su levels between patients with and without HC. **(C)** ROC curve for Su levels as a predictive marker for HC (*n* = 39). A cut off of 49.43 and 36.28 ng/mL/mg/kg for HC, before dose 7 and after dose 9, respectively [before dose 7; Area = 0.75 (95% CI: 0.59–0.93; *p* = 0.02); after dose 9; Area = 0.74 (95% CI: 0.57–0.91), *p* = 0.03]. **(D,E)** Cumulative incidences HC. Patients with HC/total number of patients in each group. **(D)** Before dose 7 **(E)** after dose 9.

We observed higher Su levels (ng/mL//mg/kg) before dose 7 (mean ± SD; 48.3 ± 11.2 vs. 35.1 ± 15.3; *p* = 0.02) and after dose 9 (40.5 ± 9.6 vs. 30.9 ± 14.4; *p* = 0.03) in patients with HC (*n* = 8) compared to those without HC (*n* = 31, **Figures 2A,B**), respectively. Cutoff values of 49.44 and 36.28 were chosen in ROC analysis for Su levels before dose 7 and after dose 9, respectively, with better sensitivity and specificity for HC occurrence (**Figure 2C**). Patients with Su levels above the cutoff value (before dose 7, 41.7%; after dose 9, 42.9%) had higher incidence of HC compared to those below the cutoff (before dose 7, 11.0%; after dose 9, 8.0%) at both dose levels (**Figures 2D,E**). Age and weight were also correlated with the incidence of HC, with higher incidence in patients older than 10 years, and weighed above 30 kg (defined by ROC curves, Supplementary Figures S2A–C). Higher levels of Su correlated with the weight and age of the patients. In multivariate analysis with Su levels and age, Su levels before dose 7 (HR: 4.6; 1.1–19.4; *p* = 0.03) and after dose 9 (HR: 6.6; 1.3–32.8; *p* = 0.02) were independently associated with HC.

### CYP2C9, GSTM1 Genotypes and HC Occurrence

**FIGURE 3:**
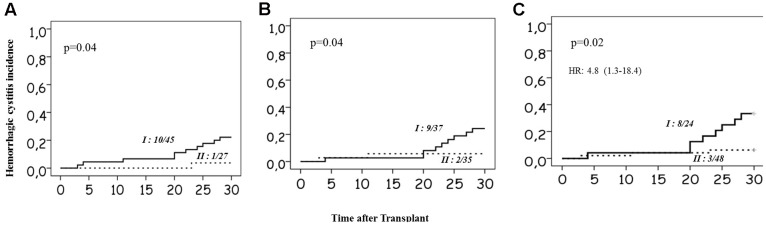
Incidence of Hemorrhagic cystitis. **(A)** in relation to the *CYP2C9* genotype status. Group I represents patients without dysfunctional alleles, and II represents carriers of at least one dysfunctional allele. **(B)** In relation to *GSTM1* genotype, group I represents nonNull carriers; and group II represents Null allele carriers. **(C)** In relation to combined *CYP2C9* and *GSTM1* genotypes group I represents carriers of normal functional *CYP2C9* and *GSTM1* genotypes and group II represents carriers of at least one dysfunctional allele in either *CYP2C9* or *GSTM1* or both. Number of patients with HC/total number of patients in each group and *p*-values are mentioned on the plots. Multivariate Cox-regression *p*-value and hazards ratio with 95% confidence intervals are mentioned for 3C.

The minor allele and heterozygosity frequencies of the genetic variants studied are given in **Table 1**. Higher incidences of HC were observed in *CYP2C9 ^∗^1^∗^1* (22.2%), *GSTM1* nonNULL genotype carriers (24.3%) and in patients carrying both functional *GSTM1* and *CYP2C9* genotypes (33.3%) compared to those carrying non-functional allele in either one of the genes (6.3%; **Figures 3A–C**). No significant associations between HC and other SNPs investigated were noted. In multivariate analysis including age, combined *GSTM1* and *CYP* genotype status was independently associated with HC with an HR of 4.8 (95% CI: 1.3–18.4; *p* = 0.02; **Figure 3C**). Su levels differed significantly between children who are above and below 10 years of age (Supplementary Figures S3A,B). We observed a trend of higher Su levels in children carrying both normal *CYP2C9* and *GSTM1* genotypes compared to those carrying either one or both dysfunctional genotypes (Supplementary Figures S3C,D). However, Su levels did not differ significantly between these two genotype groups (Supplementary Figures S3C,D).

### Effect of Su and Ac on HUC Cell Viability

Su did not affect the cell viability of HUC cells at concentrations up to 1 mM ( = 120.2 μg/mL; Supplementary Figures S4A,B). Ac was found to affect the morphology and viability and attachment of cells at concentrations above 33 μM when incubated for short time (Supplementary Figures S4A,B). The IC50 value for Ac in HUC cells was found to be 516 μM = 28.9 μg/mL (*R*^2^ = 0.92; degrees of freedom 21). Incubation of HUC with Ac for 1 h proved to be toxic and cells were apoptotic and morphology represented shrinking, round cells with fragmented nuclei. Cells started detaching from the surface following growth arrest with no live cells seen attached after 48 h. Ac showed higher binding of annexin V and increased apoptosis in HUC cells at 33 μM = 1.8 μg/mL and 50 μM = 2.8 μg/mL (Supplementary Figure S5A) and Su did not show any increase in apoptosis at concentrations lower than 1 mM (Supplementary Figure S5B).

### Su and Ac on HepG2 Cell Lines

Su was not found to be toxic in HepG2 cell lines at concentrations up to 1 mM (Data not shown). Ac was toxic to HepG2 cell lines at concentrations as low as 84 μM = 4.7 μg/mL and LD50 value for Ac in HepG2 cell lines was found to be 325 μM = 18.2 μg/mL (Data not shown). The cells were shrunk and showed apoptotic changes after incubation with Ac up to 24 h (Data not shown).

### Su Effect on CYP Activity and Expression in HepaRG Cells

HepaRG exposed to different levels of Su did not show significant differences in CYP activity (Supplementary Figure S6). No significant differences were seen at the level of *CYP* gene expression (Supplementary Figure S7). Known inducers of CYPs like phenobarbitone and rifampicin exhibited induction unlike Su (Supplementary Figures S6, S7). Data from these experiments indicates no significant impact of Su on CYP activity and expression in *HepaRG* cells. The only exception was with CYP2B6, where it resulted in a twofold induction, only at the mRNA level (Supplementary Figure S7) but it did not exhibit increased activity when phenotyped with the probe drug for CYP2B6.

### ADH Inhibition Assays

No inhibitory effect of Su on ADH was observed. Where as known inhibitor pyrazole did inhibit ADH activity in the same experiment with an IC50 consistent with previous reports (Supplementary Figure S8).

## Discussion

The major finding of this study is the clinical association of HC with *CYP2C9* and *GSTM1* genotype status and age. In this study, we established and provided evidence for a concentration dependent toxic effect of Ac in HUC cells. We have also demonstrated that exposure of urothelium to Ac at shorter times is sufficient to cause damage to the urothelium as evidenced by the increased apoptosis in HUCs exposed to Ac for up to 30 min. These observations suggest that circulating unconjugated Ac causes deleterious effects on the endothelium of blood vessels and on capillary plexus in the submucosal layer. Previously it has been demonstrated that Ac is toxic to the pulmonary endothelial cells and bronchial epithelial cells at concentrations ranging from 25 μM = 1.4 μg/mL to 100 μM = 5.6 μg/mL (Grafstrom et al., 1988; Patel and Block, 1993; Kachel and Martin, 1994). Ac can readily form adducts with proteins as it is a soft electrophile and participates in Michael adducts formation resulting in cellular apoptosis as an end effect (Cai et al., 2009). In this study we did observed lethal effect of Ac to urinary epithelial cells at concentrations as low as 1.8 μg/mL, suggesting that circulating unconjugated Ac might also have contributed to the urinary toxicity in addition to Ac reaching the bladder. Water soluble metabolite of BU, Su was not toxic to HUC cells up to concentration of 1 mM = 120.2 μg/mL which is several fold higher than the measured concentrations in plasma after BU administration (Versace et al., 2012).

Based on these observations we hypothesize that Su serves as a marker of increased BU conjugation with glutathione (GSH) and higher CYP2C9 activity and, thus representing a population with increased CY activation. The cumulative BU doses did not differ between the patients with and without HC (**Table 2**) indicating that the variability could be at the level of Su formation from the parent drug by the CYP2C9 enzyme. We previously demonstrated the role of CYP2C9 in Su formation from BU (Uppugunduri et al., 2014). In this analysis we did observe a trend of higher Su levels in children carrying both normal *CYP2C9* and *GSTM1* genotypes, but was not statistically significant (Supplementary Figure S3). Su did not display any influence on CYP2C9, 19, 3A4, and 2B6 activities at the level of phenotyping or at the level of mRNA expression *in vitro*, supporting the hypothesis that depletion of GSH and BU action on cysteine rich proteins might be the cause for increased toxicity of CY metabolites when CY is given after BU. The formation of Su indicates the simultaneous formation of GSH analog γ-glutamyl-dehydroalanyl -glycine (EdAG) (Uppugunduri et al., 2014), another metabolite which inhibits GSH irreversibly, and also participates in increased formation of protein adducts of glutaredoxins and thus increasing oxidative stress and cellular apoptosis together with BU (Scian and Atkins, 2015). Thus Su levels might reflect these sequential events predisposing to HC.

It is well-known that the metabolite of CY, Ac is urotoxic (Kehrer and Biswal, 2000). Ac and other active metabolites of CY are eliminated by GSH conjugation catalyzed by GSTs (Conklin et al., 2009). Ac spontaneously reacts with GSH and other thiols, and the formation of such Michael adducts of Ac-GSH is catalyzed by GSTs (Berhane et al., 1994). Other mechanisms of detoxification such as conjugation with cysteine residues to form mercapturates are also possible (Ramu et al., 1995). Target specific expression of GSTs determines sensitivity of the tissues to the toxicity of agents specifically eliminated via conjugation with GSH. GSTM1 is expressed in both the liver and urinary bladder (Uhlen et al., 2015), we postulate that increased conjugation of Ac with GSH in children with GSTM1 normal function facilitates its delivery to the bladder (Hashmi et al., 1992; Ramu et al., 1995) which could directly damage the urinary bladder epithelium upon its release from GSH conjugate, initiating the pathogenesis of HC. However, In support of this hypothesis, we could not measure either plasma or the urinary Ac levels from these children due to the non-availability of samples, and due to the retrospective nature of this study. On the other hand, inhibition of DNA repair process and downregulation of nuclear factor erythroid 2–related factor 2 (Nrf-2) by Ac (Qin et al., 2016) would contribute to its toxicity and of other active metabolites of CY such as phosphoramide mustard. There is also the possibility of GSH depletion in children with normal GSTM1 function when they receive BU especially in children older than 4 years of age (Ansari et al., 2013), which might also increase the toxicity of Ac when CY is administered after BU as reported recently by our group in a multicenter study (Ansari et al., 2017).

It is demonstrated that the rate of Ac appearance in urine is more crucial for HC development, rather than the cumulative exposures of Ac (Al-Rawithi et al., 1998). Inter-individual variability in the rate of Ac appearance, could be partly explained by CYP2C9 function due to genetic variations. Blood Ac levels were reported to reach up to 10.2 μM = 600 ng/mL in patients receiving 60 mg/kg of CY (Ren et al., 1999). Urinary Ac levels can reach up to 406.8 nM = 23 ng/mL and concentrations vary depending upon the urine volume. Furthermore, the time required for peak urinary levels vary (from 1 to 12 h) among individuals (Takamoto et al., 2004). In our study all children received MESNA. However, patients with normal CYP2C9, GSTM1 activity and older than 10 years of age with viruria may need alternate prophylactic measures possibly because of the increased rate of Ac formation and its increased delivery to the bladder. We did not measure urinary levels of either Su or Ac to provide a conclusive evidence for this hypothesis. Higher urinary levels of Su and Ac were assumed if higher plasma levels were observed due to their water soluble nature. A recent population pharmacokinetic modeling with genetic covariates showed that *CYP2C9* genotypes partly explained the variability in CY pharmacokinetics (Balasubramanian et al., 2012). It is possible that an increased rate of Ac formation is due to the normal function of CYP2C9. However, a combined influence of various CYP isoforms in CY metabolism should not be ignored, especially those of CYP2B6 and CYP2C19. However, in this study we did not find an association of HC with the genetic variants analyzed in these two genes.

In addition to the genotypes, age was significantly associated with HC. In this cohort children older than 10 years of age had higher HC incidences. It is known that primary BK virus infection occurs at a younger age and at 10 years of age 50% of the population are seropositive (Hirsch and Steiger, 2003; Silva Lde et al., 2010). In our cohort 73% of the children with HC had BK viruria, and 9% had JC viruria at the time of HC diagnosis indicating a predominant role of viral infection in HC etiology, with contribution of other factors such as damage by CY metabolites, increased viral replication due to poor immune reconstitution, immunosuppression. However, we do not have viral infection data from the patients without HC to attribute its etiology unconditionally to viruria. Observations from this cohort indicate that in pediatric patients receiving BU-CY regimen prior to allogeneic HSCT age, viral infection status, *CYP2C9* and *GSTM1* genotype status are important predictors of HC occurrence. These risk factors could be added to the existing risk factors to develop a risk score for patient stratification for the implementation of effective prophylactic measures. However, this study is limited by its retrospective nature including patients recruited over a prolonged time period of more than 10 years. The changing practices over time and heterogeneity of the patient population included are the other limitations of this study. No differentiation of early or late onset of HC was considered in this study, in fact we investigated HC occurrence throughout the engraftment period which on average is defined as 30 days post HSCT owing to the two different sources, i.e., cord blood and bone marrow used in our cohort. This time frame allowed us to evaluate the genetic association with consideration of the degree of immunosuppression, viral impact, aGVHD influence, donor type, and immune reconstitution. The genetic association observed in this study should be interpreted in view of the following limitations: (a) the small sample size especially for Su level measurements, (b) dearth of data on the impact of Su on enzymes such as GSTs and ALDH. (c) Lack of data on the plasma or urinary Ac levels from these children. Overall, findings of this study strongly encourage further investigation into the impact of Su on the function of enzymes other than CYPs in the metabolic pathways of both BU and CY. Further investigations are also warranted to assess the relationship between (i) Su and Ac levels, (ii) *CYP2C9, GSTM1* genotype status, Ac levels and HC in a larger set of samples.

We did observe a trend of worse event-free survival (54.5 vs. 70.5%; *p* = 0.2) and OS (64 vs. 84%; *p* = 0.12) in patients who developed HC (data not shown). Irrespective of whether HC affects survival or not, it is associated with morbidity and long-term hospitalization which could be minimized with prior identification of high risk patients. In high risk patients CY may be replaced by fludarabine, thus reducing the risk of infections and toxicity with similar clinical outcomes provided the patient has a disease with lower risk of relapse and a matched related donor is available (Ben-Barouch et al., 2016). Moreover, the preventive measures and treatment options for HC are not optimized, stratifying patients could aid in evaluating the choice of treatment and preventive measures prospectively, for example, prolonging hyperhydratation, use of prostaglandin derivatives, chemokine antagonists, use of bacterial or antiviral prophylaxis in high risk patients.

## Conclusion

High risk patients for HC can be identified based on the criteria of: older than 10 years of age, carrying normal *CYP2C9* and *GSTM1* genotypes, and having viral infections. This particular group shall be selected for evaluating different preventive measures to avoid morbidity and long term hospitalization due to HC. Ac is toxic to urothelial cells at concentrations of 33 μM. Su is not toxic to urothelial cells and does not affect the CYP function. Confirmation of this genetic association in an independent cohort is essential before it can be incorporated along with other known risk factors to develop a risk score for patient stratification in clinical care.

## Author Contributions

CU, FS, FD-L, DM, and VM performed the experiments. YD and YT supervised the analytical experiments. AR, HB, and MK recorded the clinical data. AR, HB, YT, MK, and CU managed the clinical samples. CU, FS, and MA performed the data analysis. CU, MA, and MK designed the research. CU and FS drafted the article. All authors contributed to the interpretation of data, and revised the manuscript.

## Conflict of Interest Statement

The authors declare that the research was conducted in the absence of any commercial or financial relationships that could be construed as a potential conflict of interest.

## Footnotes

1 www.graphpad.com
