# Supplementary material for: The Association of Combined GSTM1 and CYP2C9 Genotype Status with the Occurrence of Hemorrhagic Cystitis in Pediatric Patients Receiving Myeloablative Conditioning Regimen Prior to Allogeneic Hematopoietic Stem Cell Transplantation
Source: Front Pharmacol. 2017 Jul 11;8:451. doi: 10.3389/fphar.2017.00451 (PMC5504863; doi:10.3389/fphar.2017.00451)
Supplement: Supplementary file 1 [file Presentation_1.pdf]

Supplementary Material

The Association of Combined *GSTM1* and *CYP2C9* Genotype Status with the Occurrence of Hemorrhagic Cystitis in Pediatric Patients Receiving Myeloablative Conditioning Regimen Prior to Allogeneic Haematopoietic Stem Cell Transplantation.

Chakradhara Rao S Uppugunduri,[1,2] Flavia Storelli,[3] Vid Mlakar, [1,2] Patricia Curtis, [1,2] Aziz Rezgui,[4] Yves Théorêt, [5] Yves Chalandon,[6] Peter Bader,[7] Youssef Daali, [2] Henrique Bittencourt, [8] Maja Krajinovic,[4,5,8] and Marc Ansari[1,2] on behalf of Pediatric Diseases Working Party of European Society for Blood and Marrow Transplantation (PDWP-EBMT).

[1]Onco-Hematology Unit, Department of Pediatrics, University Hospital of Geneva, Geneva, Switzerland.

[2]CANSEARCH Research Laboratory, Department of Pediatrics, Faculty of Medicine, University of Geneva, Geneva, Switzerland.

[3] Clinical Pharmacology and Toxicology Service, Geneva University Hospital, Geneva, Switzerland.

[4]CHU Sainte-Justine Research Center, Charles-Bruneau Cancer Center, Montreal, Canada.

[5]Clinical Pharmacology Unit, CHU Sainte-Justine, Montreal, Canada.

[6] Division of Hematology, Department of Medical Specialties, Geneva University Hospital, Geneva, Switzerland.

[7]Division for Stem Cell Transplantation and Immunology; University Hospital Frankfurt, Frankfurt, Germany.

[8]Department of Pediatrics, Charles-Bruneau Cancer Center, CHU Sainte-Justine Research Center, Montreal, QC,Canada.

Correspondence: Prof. Dr. Marc Ansari or Chakradhara Rao S Uppugudnuri, Onco-Hematology Unit, Department of Pediatrics, University Hospital of Geneva, Geneva, Switzerland. Tel: +41 79 55 36 100, Fax: +41 22 382 31 00, E-mail: marc.ansari@hcuge.ch or chakradhara.uppugunduri@unige.ch

Supplementray Table S1: List of genes and specific primers used for gene expression analysis along with efficiencies tested

Gene	Forward primer (5’-3’)	Reverse primer (5’-3’)
CYP2C9	CTA TCT CAT TCC CAA GGG CAC AAC	TTG TCA TGT AGC ACA GAA GTC AGG
	GCC TGA AAC CCA TAG TGG TG	GGG GCT GCT CAA AAT CTT GAT G
CYP2C19	TCC AGA GAT ACA TCG ACC TCA TCC	AAT ATG GTT GTG CCC TTG GGA ATG
	TCC AGA GAT ACA TCG ACC TCA TCC	AAT ATG GTT GTG CCC TTG GGA ATG
CYP2B6	TCT GGC CGG GGA AAA ATC G	GGT CAC AGA GAA TCG CCG AAG
CYP3A4	ATT CAG CAA GAA GAA CAA GGA CA	TGG TGT TCT CAG GCA CAG AT
	CAC GAG CAG TGT TCT CTC CTT	CAC AGT ATC ATA GGT GGG TGG T
SLC25A27	TCA GGC TGT TCA AGG TGA AG	AAG CCA GAA CAC CAT TGA CC
	AAG GCT TTT TAC CAT CTT GGC	GGG TTG CAT CTT TAG GGG TT
GAPDH	TGT TGC CAT CAA TGA CCC CTT	CTC CAC GAC GTA CTC AGC G

Efficiencies of 3A4, 2B6, 2C9 are in the range of 95-102 % with CYP2C9 having efficiency of 98 % for set 1 primers and 98.4 % efficiency for the second set of primers, and 2B6 had 101 %, and 3A4 had 93 % set 1 and 97% for set 2efficiencies when tested similar to that of GAPH in 400 nM range.

Supplementary Table S2: Transitions for MS/MS analysis of metabolites

Analyte	Mode	Transition	Internal standard
**Hydroxymidazolam**	+	342.00 → 324.00	alpha-hydroxymidazolam-d4
**Hydroxybupropion**	+	256.19 → 237.89	Hydroxybupropion-d6
**Hydroxyflurbiprofen**	-	259.01 →214.87	Paracetamol-d4
**Hydroxymephenytoin**	-	239.90 → 189.80	Hydroxymephenytoin-d3
**Paracetamol-d4**	-	235.90 → 192.80	N.A
**Hydroxymidazolam-d4**	+	349.00 →328.00	N.A
**Hydroxybupropion-d6**	+	262.19 → 243.89	N.A
**Paracetamol-d4**	+	156.00 → 114.10	N.A

Supplementary Table S3. Demographic and transplantation characteristics of the children

Characteristics of the study group		Patients *N*	*%*
**Gender**	Male	36	50.0
	Female	36	50.0
**Ethnicity**	Caucasian	56	77.7
	Native American	3	4.2
	African-American	11	15.3
	Asian	2	2.8
**Diagnosis**			
Malignancies	AML	23	31.9
	MDS	18	25.0
	ALL	2	2.8
	Myeloproliferative syndrome	1	1.4
Non-Malignancies	Hemoglobinopathy	11	15.3
	Immunodeficiencies	9	12.5
	Hemophagocytic syndrome	3	4.2
	Metabolic disease	5	6.9
**HLA compatibility**	MUD	13	18.1
	MM – related donor	3	4.2
	MM – unrelated donor	29	40.3
	HLA identical sibling	26	36.1
**Stem Cell Source**	BM	31	43.1
	Cord Blood	41	56.9
**Serotherapy**	No	17	23.6
	ATG	55	76.4
**GvHD Prophylaxis**	CSA + steroids	38	52.8
	CSA + MTX	32	44.4
	CSA	2	2.8

	Median (Mean ±SD)	***Range***
**BM:**		
Nucleated cells ($x10^8$/Kg)/n=31)	10.8 (10.4±6.4)	0.11-23.2
CD34 cells ($x10^8$/Kg)/n=16)	0.2 (0.2±0.3)	0.00024-1.3
**Cord Blood:**		
Nucleated cells ($x10^8$/Kg)/n=74)	1.4 (1.9±2.5)	0.07-14.8
CD34 cells ($x10^8$/Kg)/n=73)	0.004 (0.016±0.033)	0.00019-0.143
**Age (years)**	6.9 (7.7±5.8)	0.1-19.9
**Weight (kg)**	24.9 (28.7±19.7)	4.3-81.1
**Height (cm)**	122.5 (117.2±35.9)	54.0-179.5

Abbreviations ALL: acute lymphoblastic leukemia; AML: acute myeloid leukemia; ATG: anti-thymocyte globulin; BM: bone marrow; CSA: cyclosporine; MDS: myelodysplastic syndrome; MM: mismatch; MRD: matched related donor; MUD: matched unrelated donor; MTX: methotrexate; CSA: cyclosporine; SD: sibling donor; GvHD: graft-versus-host-disease; PBSC: peripheral blood stem cells; All patients Received BU-CY myeloablative conditioning regimen prior to HSCT. CD34 positive cells infused data is missing for 15 patients who received BM.

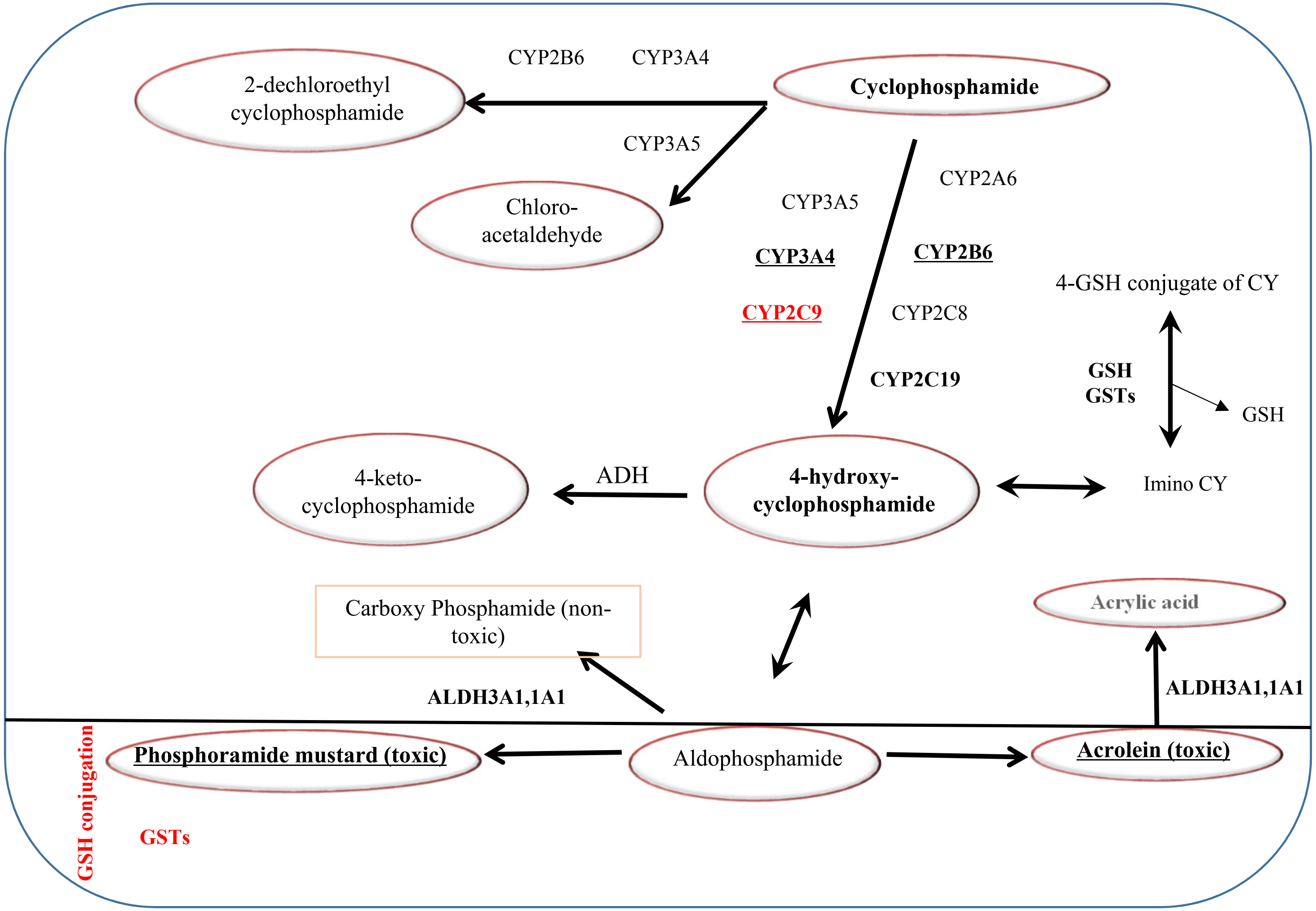

Supplementary Figure S1. Metabolic pathway of cyclophosphamide.
Cyclophosphamide undergoes metabolic activation to hydroxy cyclophosphamide catalyzed by Cytochrome P450 enzymes such as 3A4, 2B6, 2C9 and 2C19. The active metabolites of cyclophosphamide are eliminated via conjugation with glutathione (GSH), catalyzed by glutathione s transferase enzymes (GSTs). Aldehyde dehydrogenase (ALDH3A1, and 1A1) are involved in the conversion of toxic aldophosphamide to non-toxic carboxy phosphamide. Alcohol dehydrogenase enzyme (ADH) is involved in conversion of hydroxy to keto form. Phosphoramide mustard, and acrolein are the toxic metabolites of cyclophosphamide. This figure is adapted from Zhang et al., Br J Clin Pharmacol 2006; 62:457-472

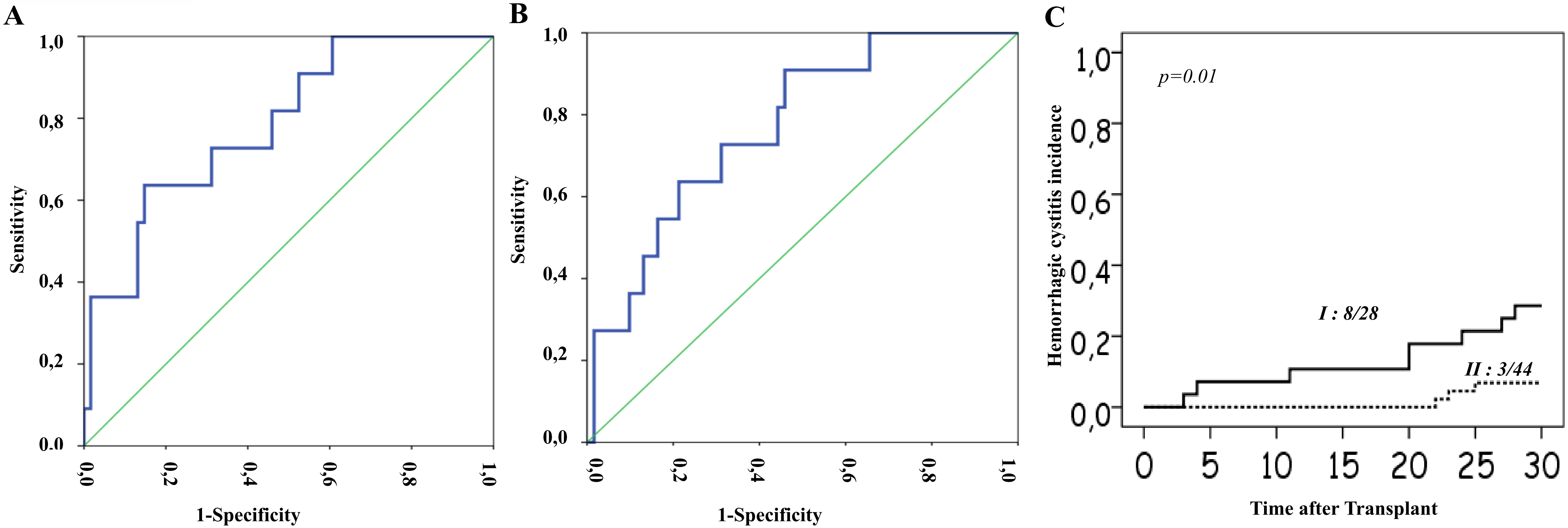

Supplementary Figure S2. ROC analysis A) with age B) with weight as predictors of HC. Area under the curve for age 0.79 (95% Confidence interval 0.65 to 0.93; p=0.003) and for weight was 0.77 (95% Confidence interval 0.63 to 0.91; p=0.004). Cutoff for age 10 years, and weight 30 kg was defined with 73 % sensitivity and 70% specificity approximately. As direct linear relationship exists between age and weight for multivariate analysis only age variable was used. **C) Cumulative incidences of HC in patients older than 10 years of age (Group I) and below 10 years of age (Group II)**. The age cut off was defined by defined by ROC analysis, the number of patients in each group with HC occurrence / total number of the patients in the group and p values are given on the plot.

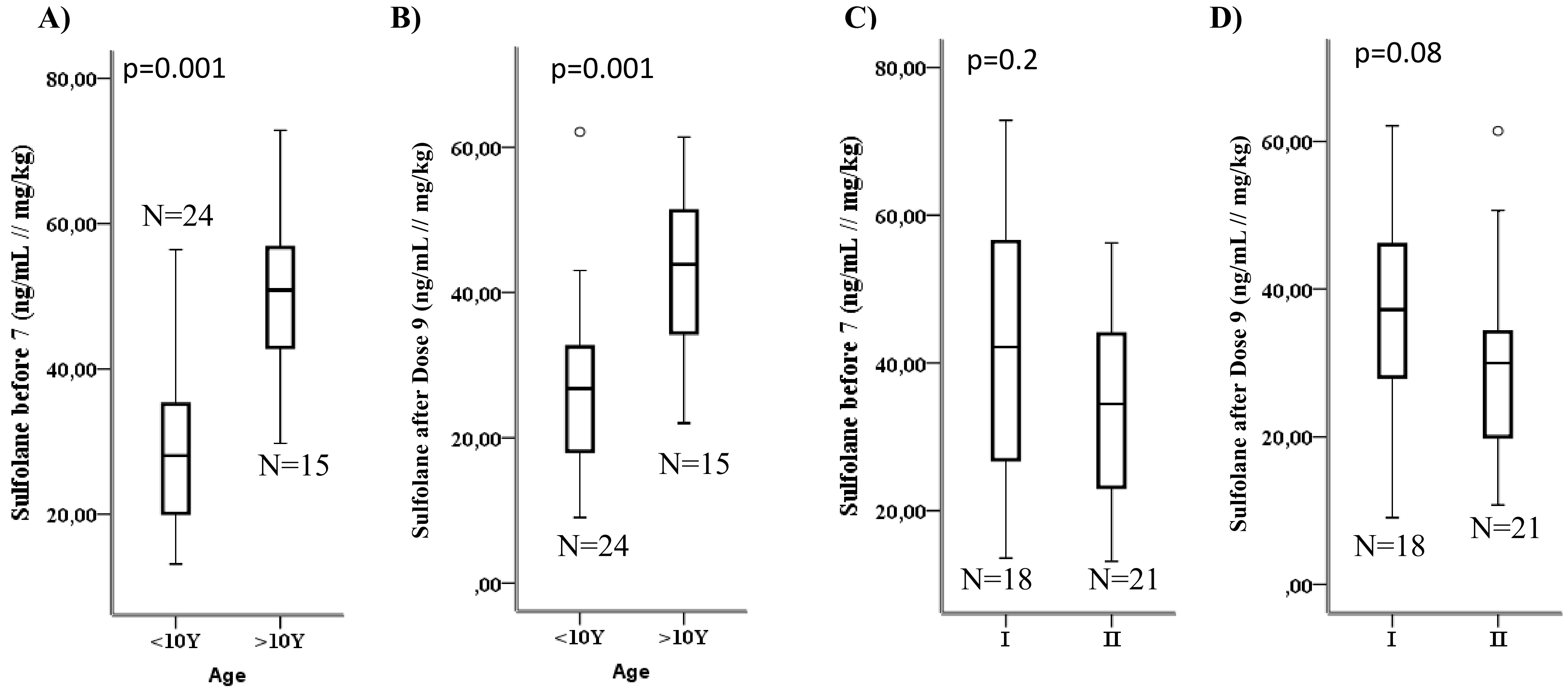

Supplementary Figure S3 . Sulfolane levels in relation to age and *CYP2C9, GSTM1* genotype status. A, B) Sulfolane levels between children below and above 10 years of age (n=72). C, D) Sulfolane levels between children carrying both normal *CYP2C9 , GSTM1* genotypes (I) and carrying either one or both dysfunctional genotypes (II). The number of children in each group and the p values are presented on the plots.

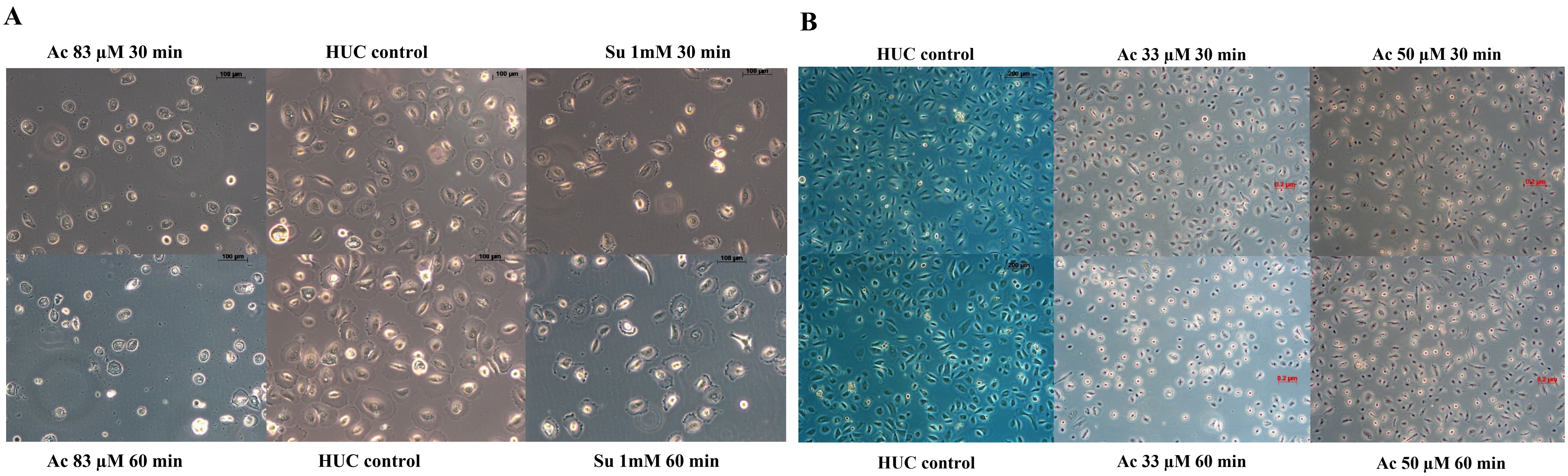

Supplementary Figure S4. Influence of Sulfolane and Acrolein short term exposures on human urothelial cell (HUC) morphology. Su did not affect the morphology of cells up to 1 mM concentration (A). A significant change to cell's and nucleus' morphology can be observed after only 30 min exposure to concentrations as low as 33 µM or 50 µM Ac (B) and the effect is prominent at 83 µM (A). The cell and nucleus rounding effect is even more pronounced after 60 min exposure.

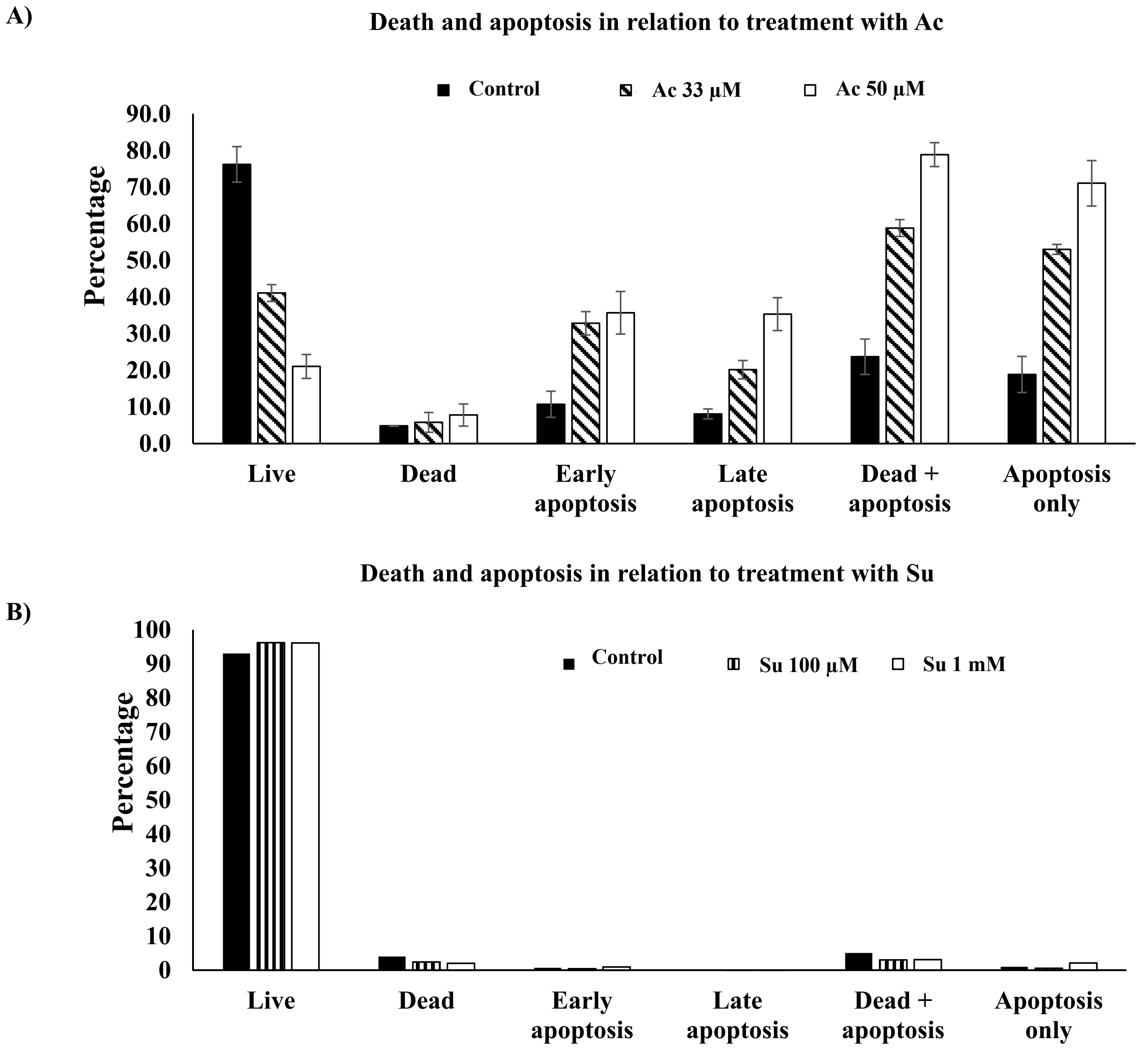

Supplementary Figure S5. **Annexin V and propidium iodide apoptosis assay results** A) Influence of Ac on HUC cells. For 33 µM t-test was performed in comparison to Control ($p<0.05$ for all comparisons). For 50 µM t-test was performed in comparison to 33 µM ($p<0.01$ for all comparisons). B) Influence of Sulfolane on HUC cells. No significant impact was observed.

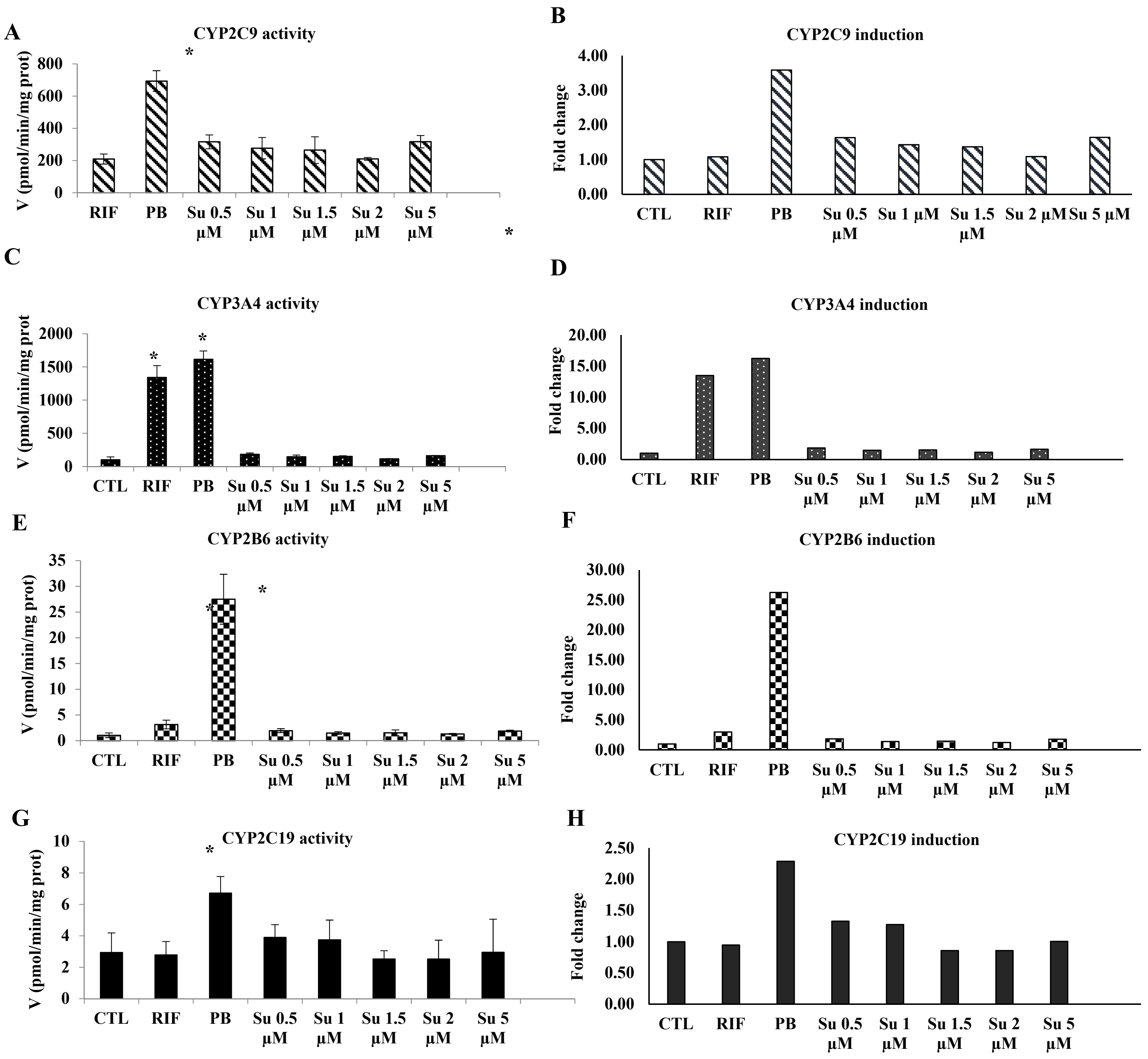

Supplementary Figure S6. CYP activity measured as metabolite formation velocity after exposures to rifampicin (RIF: 50 µM), Phenobarbital (PB: 1mM) and Su (0.5, 1, 1.5,2, and 5 µM). Su did not induce activity of the CYP2C9, 19,3A4 and 2B6 measured with the probe drugs after pre-incubation (see methodology). Phenobarbitone induced all the four enzymes tested, and rifampicin induced CYP3A4.

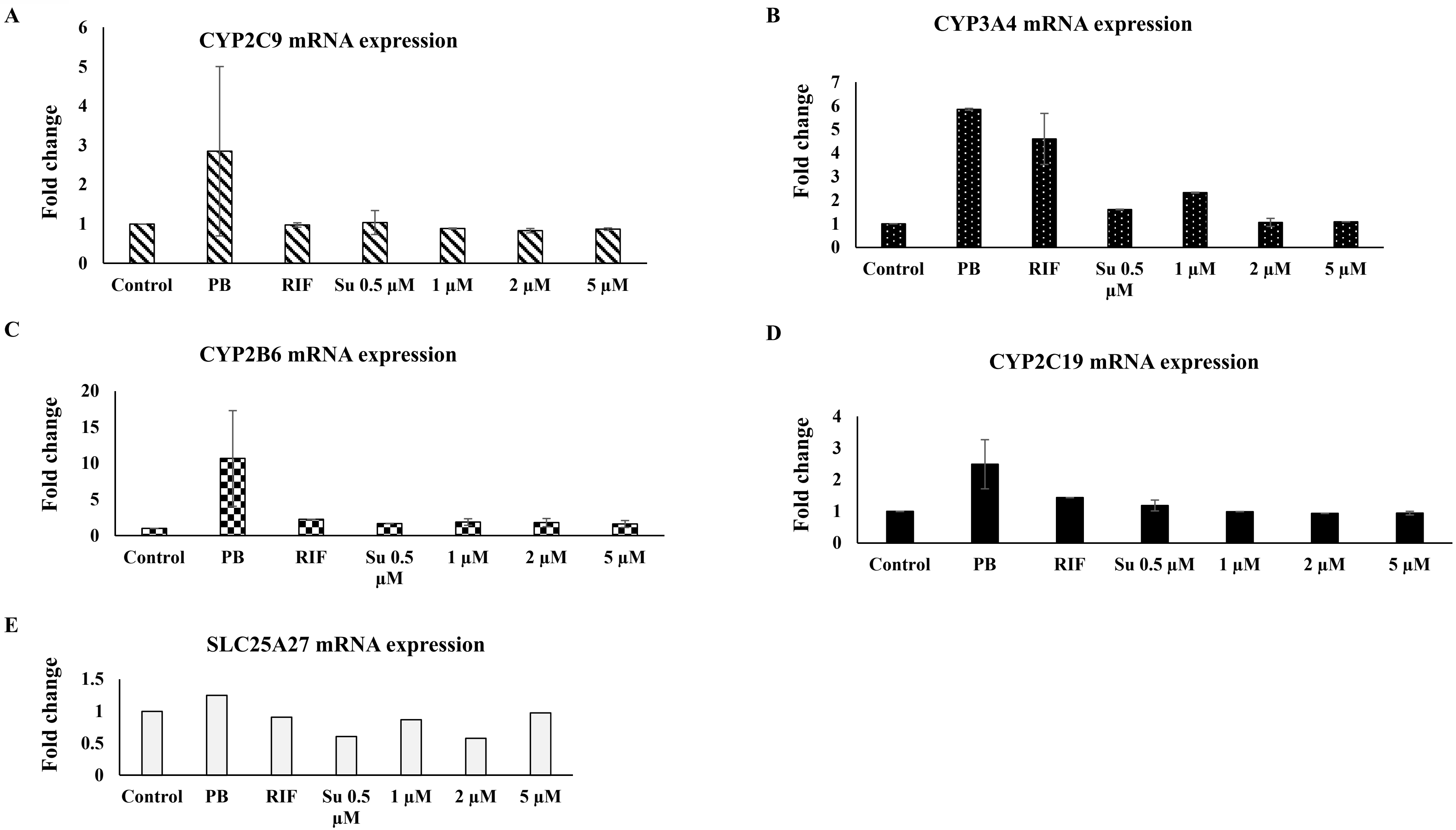

Supplementary Figure S7. Gene expression changes in HepaRG cell lines pretreated with Su prior to CYP phenotyping. Pooled cells from triplicates wells used for phenotyping and for gene expression experiments on two different occasions, and each time were performed in duplicates. Fold changes in the expression is presented compared to the control calculated from delta delta Ct method followed by fold conversion using 2^-delta delta Ct. PB: phenobarbitone; RIF: Rifampicin, Su: sulfolane.

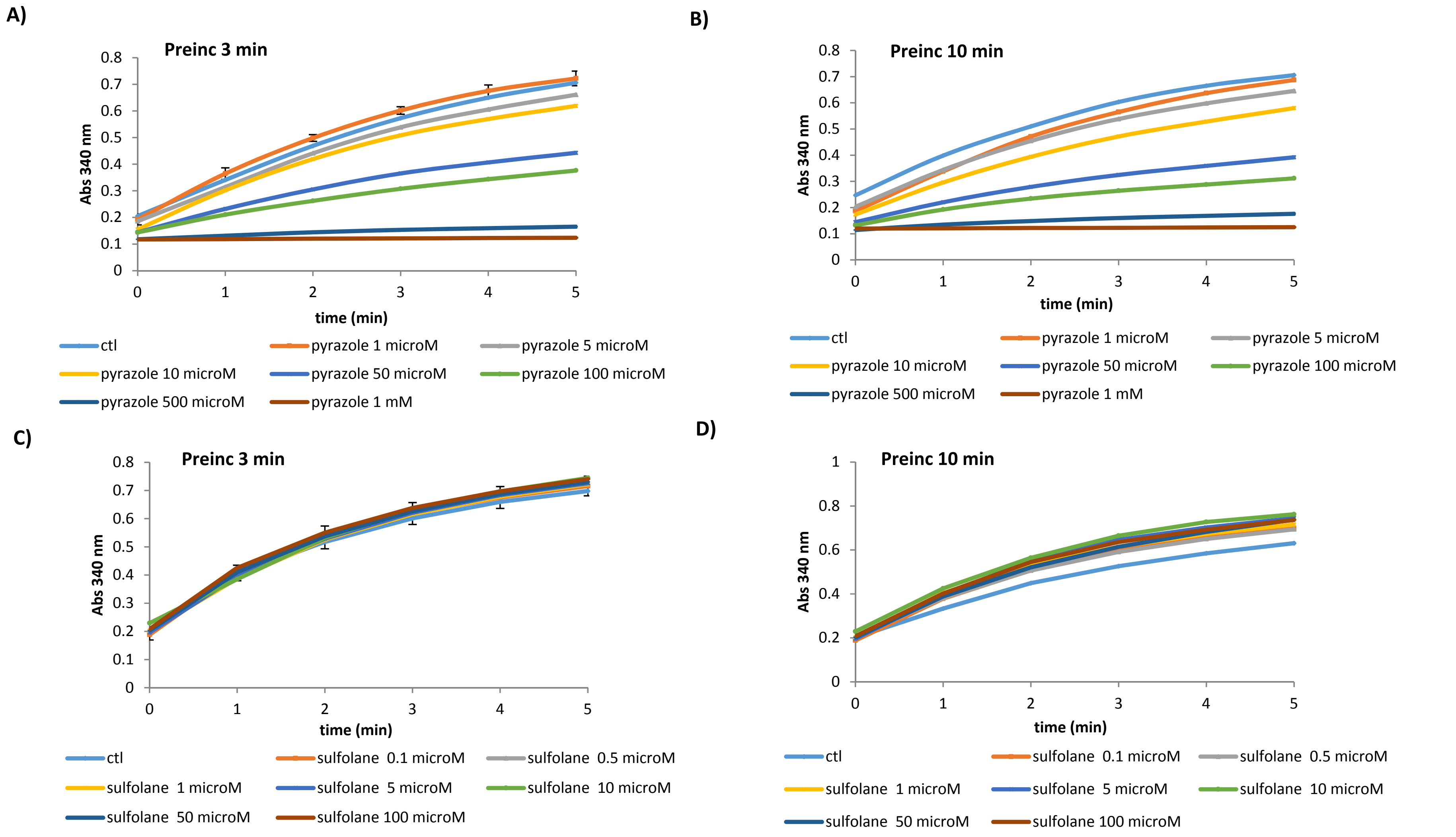

Supplementary Figure S8. **ADH inhibition assays**. A, B) pre incubation with a known ADH inhibitor pyrazole at 5 different concentrations. C, D) Pre incubation with Su at 5 different concentrations.
